# Correlates of Anxiety and Depression Among Youth in India: Findings from a Large-Scale Population-Based Cross-sectional Study

**DOI:** 10.34172/jrhs.11330

**Published:** 2025-10-18

**Authors:** Nidhi Saraswat, Pradeep Banandur, Gautham Melur Sukumar, Shalin Lily Giboy, Arvind Banavaram Anniappan

**Affiliations:** ^1^Department of Epidemiology, Centre for Public Health, NIMHANS, Bengaluru, India

**Keywords:** Youth, Depression, Anxiety, Risk factors, India

## Abstract

**Background:**

Anxiety and depression are major public health concerns due to their high prevalence and associated suffering, dysfunction, and socioeconomic impact, particularly among young individuals. Identifying factors associated with anxiety and depression is crucial for the prevention and promotion of mental health in youth. The present analysis aims to identify factors associated with anxiety and depression among youth, based on a youth health survey undertaken in Kolar district, India.

**Study Design:**

This study employed a cross-sectional design.

**Methods:**

A secondary data analysis was conducted using data from the Kolar Youth Health Survey, which collected data on various health-related behaviors and conditions from 5,072 youth (aged 15–30 years). Anxiety and depression were screened using standardized tools (GAD-7 and PHQ-9, respectively). Multivariable logistic regression was conducted to identify associated factors. The model’s goodness of fit was evaluated using the Hosmer-Lemeshow test and the area under the curve.

**Results:**

Socio-demographic characteristics (marital status, taluka, age), self-reported diagnosed health conditions, sleep issues, suicide and non-suicidal self-harm, tobacco dependence, time spent on phone/computer, family relationships (loving/affectionate relationships vs. serious conflicts), friendships, belief in God, and injury (physical violence, road traffic injuries) were significantly associated with anxiety and/or depression among youth in the study area.

**Conclusion:**

Selected sociodemographic characteristics, health-impacting behaviors, and health issues were found to be significantly associated with anxiety and/or depression among youth. Considering these risk factors will enable health care providers and policymakers to develop and implement tailored interventions.

## Background

 Globally, years lived with disability (YLDs) due to anxiety disorders alone account for 3.34% of the total YLDs.^1^ The National Mental Health Survey of India (NMHS) estimates the overall prevalence of generalized anxiety disorders at 0.6% among adults.^2^ Similarly, depression is a major public health concern affecting 4.4% of the global population.^3^ According to NMHS, the current prevalence of depressive disorders among adults in India is 2.7%, with 1.6% among the 18-29 age group.^2^ Anxiety and depression are major public health concerns due to their high prevalence and their association with suffering, dysfunction, and socioeconomic impact, particularly among young individuals undergoing rapid social, emotional, and cognitive development changes.

 Depression and anxiety in young people are often linked to factors such as academic challenges, financial difficulties, family conflicts, substance abuse, poor nutrition, inadequate sleep, sedentary behavior, and excessive screen time. ^4-7^ Additional risk factors include childhood trauma, higher body mass index (BMI), and chronic health conditions.^8^ Identifying and understanding these causes and risk factors is crucial for preventing these mental health issues and promoting overall well-being in youth. Early recognition and timely intervention are key to minimizing both immediate and long-term effects of mental disorders, improving outcomes, and alleviating the associated socioeconomic burden.^9^ Focusing on prevention and mental health promotion among youth is essential, as anxiety and depression can profoundly impact overall well-being, educational achievement, employment opportunities, and economic productivity.^8^ This emphasis aligns with the Sustainable Development Goals (SDGs), particularly those aimed at enhancing health and well-being and fostering inclusive communities.

 As rates of depression and anxiety continue to rise, there is an urgent need for research to identify factors associated with these conditions among youth and to design effective interventions based on the findings. However, such research remains limited in the Indian context. Previous studies have attempted to examine factors associated with anxiety and depression only among subsets of the youth population, such as adolescents or school/college-going students. Addressing these limitations, the present study aimed to assess the factors associated with anxiety and depression among youth aged 15-30 years, using data from a comprehensive youth health survey conducted in Kolar district, India.

## Materials and Methods

 The Centre for Public Health, Department of Epidemiology, NIMHANS, undertook the comprehensive Kolar Youth Health Survey in Kolar district, Karnataka, in 2022. A total of 5072 youth (sample size estimated following standard scientific methods) were randomly selected using a two-stage cluster sampling technique with probability proportional to population size, covering both urban and rural areas across all six talukas of Kolar district, thereby ensuring complete representation of the district’s youth population. Eligible participants included youth aged 15-30 years who were permanent residents of the Kolar district. Data were collected through interviews and covered a wide range of health-impacting behaviors and health conditions of public health importance, including screening for depression and anxiety.

 The present study, a secondary data analysis, was conducted in 2023 using individual-level data collected from 5072 youth. Data were available on the following parameters: sociodemographic information, nutrition, physical activity, tobacco, alcohol, drug use, sexual behavior, gambling, violence, depression,anxiety, suicide, non-suicidal self-harm, road-use behavior, technology use, sleep, health and health seeking behavior, communication issues with parents, peer network, leisure-time activities, volunteerism, reading habit, spirituality, and anthropometric measurements.

 Anxiety and depression were the primary outcome measures of interest. Both were assessed using the Generalized Anxiety Disorder-7 (GAD-7) and Patient Health Questionnaire-9 (PHQ-9) scales. The GAD-7 and PHQ-9 are self-report tools used to screen for anxiety and depression, respectively. GAD-7 is a 7-item questionnaire that assesses the frequency of symptoms over the past two weeks, including nervousness, excessive worrying, fear of something bad happening, irritability, and difficulty relaxing. Responses range from 0 (not at all) to 3 (nearly every day), with total scores between 0 and 21 and higher scores indicating more severe anxiety. The PHQ-9 is a 9-item questionnaire measuring depressive symptoms over the last two weeks, such as lack of interest or pleasure in activities, feeling down or hopeless, difficulty sleeping, low energy, poor appetite, feelings of worthlessness, difficulty concentrating, and thoughts of self-harm or suicide. Each item is scored from 0 (not at all) to 3 (nearly every day), with total scores ranging from 0 to 27. Higher scores indicate more severe depression. In the present study, individuals scoring 10 or more on either scale were considered to have screened positive for anxiety and depression.^10,11^

**Figure 1:**
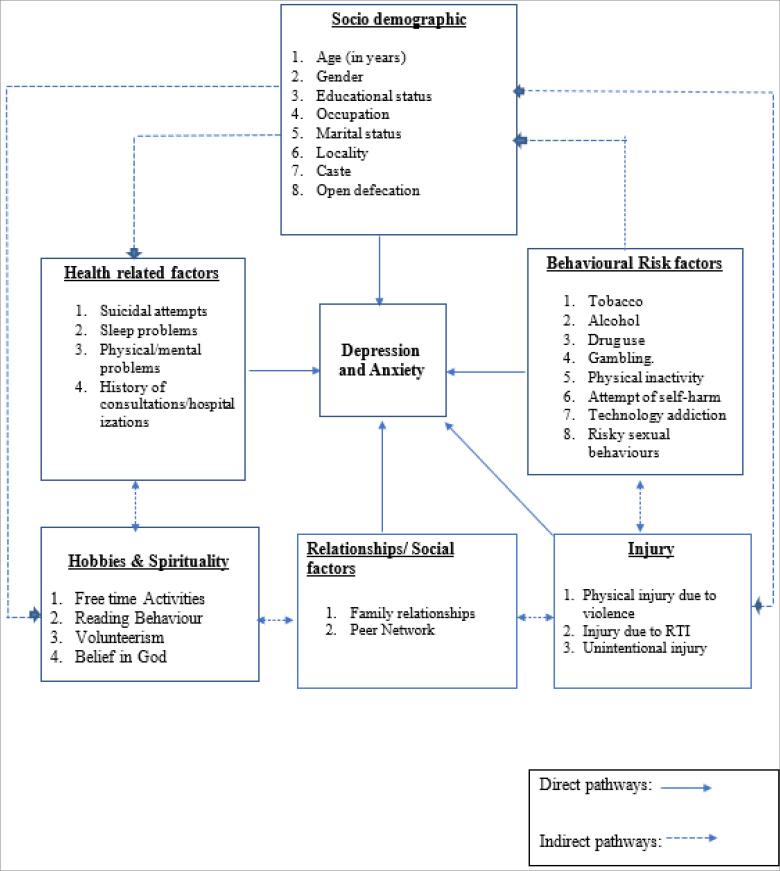
Conceptual framework showing factors associated with anxiety and depression among youth

 Based on the literature review, a conceptual framework of hypothesized exposure variables was developed for anxiety and depression (Figure 1). Broadly, these include sociodemographic characteristics, risk factors, health conditions, health-seeking behaviors, relationships, hobbies, and spirituality. All these factors are interrelated, and their interactions can in turn influence anxiety and depression.

 The dataset acquired from the project team was checked for completeness and cleaned for errors. De-identified data of 5072 participants, with all individual identifiers removed, were used for analysis. Data transformation was carried out by recoding variables wherever necessary. Univariate analysis was performed first, followed by multivariable analysis for anxiety and depression outcomes independently. All hypothesized exposure variables significantly associated with the outcome at a 10% level (*P* < 0.10) in univariate analysis were considered for inclusion in the final model using the stepwise logistic regression method. Variables significant at the 5% level (*P* < 0.05) were retained in the final model. Model fit was assessed using the Hosmer-Lemeshow test, and predictive accuracy was evaluated using the area under the curve (AUC) from ROC analysis. The data analysis was performed using SPSS version 25.

 In the primary study, written informed consent was obtained from all participants. For those aged 15–18 years, assent was obtained along with parental or guardian consent, as appropriate. The informed consent process was fully explained, including the study’s purpose, objectives, methodology, and the right to refuse participation without any consequences.

## Results

 As illustrated in Table 1, the sociodemographic profile of the study population indicated that 39.7% of youth were in the 15–20-year age group, and females constituted 53.8% of the sample. The urban and rural distribution was 32.5% and 67.5%, respectively. In terms of occupation, 39.1% of the youth interviewed were students, 25.7% were homemakers, and 35.1% were engaged in various occupations. Regarding education, 39.2% had completed upper primary/high school, while 29.4% had completed degree/diploma/ITI/postgraduate qualifications. Most participants (64.7%) were never married, while 0.9% were widowed, divorced, or separated.

**Table 1:** Sociodemographic characteristics of the study population (N = 5072)

**Characteristics **	**Number**	**Percent**
Age (y)		
15-20	2015	39.7
21-25	1603	31.6
26-30	1454	28.7
Sex		
Male	2342	46.2
Female	2730	53.8
Taluka/Sub-district		
Bangarpete	630	12.4
KGF	908	17.9
Kolar	1271	25.1
Malur	794	15.7
Mulabagal	862	17.0
Srinivasapura	607	12.0
Locale		
Urban	1648	32.5
Rural	3424	67.5
Occupation		
Business/administrative/executive/managerial/clerical	486	9.6
Agricultural/non-agricultural laborer/salesperson/shop worker	705	13.9
Skilled Manual/Machinery	503	9.9
Homemaker	1305	25.7
Student	1983	39.1
Unemployed/others	90	1.8
Education		
Not literate/lower primary (class 1-5)	157	3.1
Upper primary (class 6-7)/high school (class 8-10)	1990	39.2
Higher secondary/PUC (class 11-12)	1433	28.3
Degree/Diploma/ITI/ post graduate and above	1492	29.4
Marital status		
Currently married	1748	34.5
Never married	3280	64.7
Widowed/divorced/separated	44	0.9

*Note.* KGF: Kolar Gold Fields; PUC: Pre-university course; Taluka: Sub-district administrative division.

 As shown in Table 2, youth who were divorced, separated, or widowed had a 19-fold higher risk of screening positive for anxiety compared to those currently married (AOR = 19.19; 95% CI: 6.37-57.79). Smoking dependence was associated with nearly a fourfold higher odds of being screened positive for anxiety (AOR = 4.76; 95% CI: 1.07-21.28) compared to those without smoking dependence. Youth reporting injuries from physical violence in the past 12 months had three times higher odds compared to the reference category (AOR = 3.82; 95% CI: 1.45-10.08), while those with road traffic injuries (RTIs) were twice as likely to be screened positive for anxiety (AOR = 2.22; 95% CI: 1.06-4.63). The likelihood of being screened positive for anxiety was found to be 10 times higher among those who screened positive for depression (AOR = 10.47; 95% CI: 6.15-17.82). Similarly, sleep problems in the past 30 days increased the odds 10-fold (AOR = 10.30; 95% CI: 3.93-27.00) compared to those without sleep problems, and non-suicidal self-injury (NSSI) in the past year doubled the odds (AOR = 2.50; 95% CI: 1.26-4.95) compared to the reference category. Those diagnosed with chronic health conditions had a fivefold increased risk (AOR = 5.12; 95% CI: 2.19-11.96) compared to the reference group. Additionally, youth who self-reported poor mental health in the past 30 days were six times more likely to screen positive for anxiety (AOR = 6.72; 95% CI: 3.45, 13.10) compared to those without mental health issues during the same period.

**Table 2:** Multivariable logistic regression analysis showing factors associated with anxiety (screened positive) among youth in Kolar district, India

**Variables**	**Unadjusted OR (95% CI)**	***P*** ** value**	**Adjusted OR (95% CI)**	***P*** ** value**
Marital status				
Currently married	Ref.		Ref.	
Never married	1.42 (0.89, 2.25)	0.142	1.16 (0.67, 2.03)	0.595
Widowed/separated/divorced	20.27 (9.04, 45.48)	0.001	19.19 (6.37, 57.79)	0.001
Smoking dependence				
No	Ref.		Ref.	
Yes	15.73 (5.04, 49.10)	0.001	4.76 (1.07, 21.28)	0.041
Injured in physical violence				
No	Ref.		Ref.	
Yes	10.00 (5.09, 19.66)	0.001	3.82 (1.45, 10.08)	0.007
Depression				
No	Ref.		Ref.	
Yes	53.17 (34.17, 82.75)	0.001	10.47 (6.15, 17.82)	0.001
Non-suicidal self-injury				
No	Ref.		Ref.	
Yes	15.10 (9.24, 24.67)	0.001	2.5 (1.26, 4.95)	0.009
Road traffic injury				
No	Ref.		Ref.	
Yes	5.9 (3.42, 10.16)	0.001	2.22 (1.06, 4.63)	0.034
Time spent on computer	1.00 (1.00, 1.00)	0.001	1.00 (1.00, 1.00)	0.003
Sleep Issues				
No	Ref.		Ref.	
Yes	27.27 (11.93, 62.36)	0.001	10.3 (3.93, 27.00)	0.001
Self-reported diagnosed health issues				
No	Ref.		Ref.	
Yes	5.96 (3.17, 11.21)	0.001	5.12 (2.19, 11.96)	0.001
Poor mental health in past 30 days				
No	Ref.		Ref.	
Yes	29.07 (16.17, 52.26)	0.001	6.72 (3.45,13.10)	0.001
Affectionate relationship with family				
Very great extent	Ref.		Ref.	
Large extent	2.25 (1.48, 3.41)	0.001	0.86 (0.51, 1.45)	0.576
Some extent/not at all	3.16 (1.58, 6.32)	0.001	0.36 (0.15, 0.89)	0.026
Serious conflicts with family				
No	Ref.		Ref.	
Yes	6.77 (4.49-10.21)	0.001	1.89 (1.09, 3.27)	0.023

*Note.* OR: Odds ratio; CI: Confidence interval. Diagnosed health issues include hypertension, diabetes, stroke, cardiac disorders, thyroid, and cancer.

 Every unit increase in time spent on computer was associated with a slight increase in the risk of anxiety (AOR = 1.00; 95% CI: 1.00-1.00). Surprisingly, youth who reported only some or no affectionate family relationships had a 64% lower risk of anxiety compared to those with very affectionate family relationships (AOR = 0.36; 95% CI: 0.15-0.89). However, participants with serious family conflicts in the past 12 months had 1.8 times higher odds of anxiety (AOR = 1.89; 95% CI: 1.09-3.27). The goodness of fit revealed that the model was a good fit (AUC = 0.964; Hosmer- Lemeshow χ^2^ = 2.392, *P* = 0.935).

 As illustrated in Table 3, youth residing in Kolar Gold Field (KGF) taluka had twice the odds of screening positive for depression compared to the reference category (AOR KGF = 2.04; 95% CI: 1.048-3.987). Similarly, the likelihood of depression was twice as high among participants aged 26-30 years compared to their younger counterparts (AOR 21-25 = 1.21; 95% CI: 0.71-2.04, AOR 26-30 = 2.10; 95% CI: 1.25-3.52). Youth screening positive for anxiety had a nine-fold higher risk of depression (AOR = 9.40; 95% CI: 5.61-15.74) compared to the reference category. Reporting a suicide attempt in the past 12 months was also significantly associated with depression (AOR = 2.75; 95% CI: 1.32-5.71). Participants with sleep problems in the past 30 days were three times more likely to screen positive for depression (AOR = 3.75; 95% CI: 2.07-6.78) compared to their counterparts, while self-reported poor mental health in the past 30 days increased the likelihood of depression tenfold (AOR = 10.20; 95% CI: 5.85-17.77) compared to those without mental health issues.

**Table 3:** Multivariable logistic regression analysis showing factors associated with depression (screened positive) among youth in Kolar district, India

**Variables**	**Unadjusted OR (95% CI)**	***P*** ** value**	**Adjusted OR (95% CI)**	***P*** ** value**
Taluka/Sub-district				
Bangarpete	Ref.		Ref.	
Kolar Gold Fields	1.98 (1.15, 3.43)	0.015	2.04 (1.05, 3.99)	0.036
Kolar	0.96 (0.54, 1.71)	0.897	0.87 (0.44, 1.73)	0.697
Malur	0.66 (0.33, 1.31)	0.231	1.12 (0.50, 2.54)	0.780
Mulabagal	0.48 (0.23,1.00)	0.051	0.73 (0.31, 1.72)	0.474
Srinivasapura	0.57 (0.26, 1.24)	0.158	0.58 (0.23, 1.46)	0.249
Age group (y)				
15-20	Ref.		Ref.	
21-25	1.36 (0.88, 2.12)	0.168	1.21 (0.71, 2.04)	0.485
26-30	2.14 (1.42, 3.23)	0.001	2.10 (1.25, 3.52)	0.005
Physical activity (MET score)	1.00 (1.00, 1.00)	0.001	1.00 (1.00, 1.00)	0.021
Anxiety				
No	Ref.		Ref.	
Yes	53.17 (34.17, 82.75)	0.001	9.40 (5.61, 15.74)	0.001
Suicide attempts				
No	Ref.		Ref.	
Yes	13.41 (7.40, 24.31)	0.001	2.75 (1.32, 5.71)	0.007
Time spent on mobile phone	1.00 (1.00, 1.00)	0.001	1.00 (1.00, 1.00)	0.001
Sleep issues				
No	Ref.		Ref.	
Yes	14.55 (8.49, 24.94)	0.001	3.75 (2.07, 6.78)	0.001
Poor mental health in past 30 days				
No	Ref.		Ref.	
Yes	30.16 (18.29, 49.73)	0.001	10.20 (5.85, 17.77)	0.001
Affectionate relationship with family				
Very great extent	Ref.		Ref.	
Large extent	2.67 (1.84, 3.87)	0.001	1.18 (0.75, 1.83)	0.475
Some extent/not at all	7.24 (4.43, 11.83)	0.001	2.50 (1.37, 4.65)	0.004
Friends				
No	Ref.		Ref.	
Yes	0.64 (0.44, 0.93)	0.020	0.44 (0.27, 0.72)	0.001
Belief in God				
Strongly believe	Ref.		Ref.	
Believe	3.33 (1.82, 6.10)	0.001	2.64 (1.24, 5.63)	0.012
Neutral	2.50 (1.44, 4.32)	0.001	2.09 (1.08, 4.05)	0.029
Do not believe	1.44 (0.95, 2.17)	0.082	1.27 (0.78, 2.07)	0.338

*Note.* MET: Metabolic equivalent of task.

 Every unit increase in time spent on the mobile phone use was associated with a slightly higher risk of depression (AOR = 1.00; 95% CI: 1.00-1.00). Similarly, each unit increase in metabolic equivalent of task (MET) score for physical activity was linked to an increased risk of depression (AOR = 1.00; 95% CI: 1.00-1.00). Having friends appeared to reduce the odds of depression by 56% (AOR = 0.44; 95% CI: 0.27- 0.72). Youth who had a loving and affectionate relationship with their family to a great extent were less likely to test positive for depression, while those reporting less affectionate relationships had 2.5 times higher odds (AOR = 2.50; 95% CI: 1.37-4.65). Compared to participants who strongly believed in God, those who only “believed” or were “neutral” were at twice the risk of depression (AOR-believe = 2.64; 95% CI: 1.24-5.63; AOR-neutral = 2.09; 95% CI: 1.00-4.051). The model demonstrated good fit (AUC = 0.940; Hosmer- Lemeshow χ^2^ = 4.898; *P* = 0.768).

## Discussion

 The Kolar Youth Health Survey is one of the largest population-based surveys undertaken among youth in India. It comprehensively examines various health conditions and health-impacting behaviors among youth. Consequently, the present secondary study could explore the influence of diverse health and behaviors of youth on depression and anxiety, rendering it unique in the Indian context. The key takeaway from the analysis is that various sociodemographic factors, current health issues, health-impacting behaviors, injury and violence, technology use, sleep-related issues, family relationships, social networks, and spirituality were all significantly associated with anxiety and depression among youth.

 Studies underscore the intricate relationship between anxiety and depression, revealing how these disorders co-occur and influence one another through persistent thought patterns, creating self-sustaining cycles.^12^ Our study confirms this relationship among youth, finding a strong bidirectional relationship: those with one condition were far more likely to experience the other. Anxiety and depression are closely linked, often overlapping in symptoms, which highlights the importance of identifying and managing them together.^13,14^ Youth who self-reported poor mental health had a greater risk of screening positive for anxiety and depression, a notable finding in this study. This suggests that youth suffering from these symptoms may recognize and associate them with poor mental health. Despite limited evidence in the literature regarding this observation, it suggests that self-reported poor mental health among youth may serve as a practical indicator for screening anxiety and depression among youth in primary healthcare settings.

 NSSI is gradually gaining societal attention but remains understudied, particularly concerning its link to anxiety. A study on self-injuring students reported higher anxiety and stress levels, with 16.6% exhibiting probable anxiety disorder symptoms.^15^ Additionally, respondents with anxiety, or both depression and anxiety, were also significantly more likely to engage in self-harm compared to those without these disorders.^15^ Similarly, our study found that youth engaging in NSSI were considerably more likely to also experience anxiety, reflecting the role of self-directed negative emotions in this association. NSSI is common among those prone to negative self-directed emotions and self-criticism, which may help explain this association.^16^ Depression, in turn, is strongly associated with suicide. A systematic review found that 8.7% to 27.8% of individuals who died by suicide in India had concurrent depression.^17^ Our study similarly observed that youth with a history of suicide attempts were more likely to have depression, underscoring the need for close monitoring of this vulnerable group. Depression is a known precursor of suicide attempts, and symptoms may even occur after an attempt. This underscores the importance of regular follow-up of youth who have a history of suicide attempts to ensure early detection of depression and provide targeted interventions for at-risk youth.

 The relationship between technology use and mental health has been well studied, with evidence suggesting that excessive social media use is associated with adverse outcomes, including depression and anxiety, particularly among adolescents and young adults.^18^ The present study observed that greater use of computers and cell phones was linked with slightly elevated risks of anxiety and depression among youth in Kolar district. Prior research has reported that high screen time is significantly associated with anxiety,^19,20^ and there is a positive correlation between problematic phone use and depression.^21,22^ Although the observed associations in this study were not strong, the widespread prevalence of cell phone or technology use among youth could still have a significant population-level impact, making the findings clinically important. Negative digital experiences, particularly those involving social media, technology addiction, or reduced social interaction due to excessive technology use, could have contributed to the increased risk of anxiety and depression observed in this study population.

 Youth reporting sleep issues in the past 30 days were significantly more likely to experience both anxiety and depression, reinforcing evidence that poor sleep is a critical mental health risk factor. A prospective cohort study found that sleep disturbances predict increased anxiety symptoms,^23^ and other studies have linked insomnia to later development of depression.^24,25^ Insomnia can impair memory and concentration, cause mood disruption, particularly irritability, and interfere with daily activities, making individuals feel sleepy during the day. These outcomes collectively may increase the risk of anxiety and depression. Interestingly, contrary to prior studies, physical activity emerged as a risk factor for depression in this research. However, the strength of this association was very low, and since the majority of youths in the study area were physically inactive (82.7%), this may have influenced the observed results. Further research is needed to clarify the true relationship between physical activity and depression among youth in the study context.

 Interpersonal relationships hold considerable importance in an individual’s life and play a fundamental role in their overall well-being. In line with the existing literature, youth who reported serious conflict with family members at least once in the last 12 months had an increased risk of anxiety. Family disputes have an impact on multiple aspects of life, thereby elevating the risk of anxiety and other mental health conditions.^26,27^ The risk of anxiety was also higher among separated, divorced, or widowed youth compared to currently married youth, aligning with existing evidence.^28-30^ In our study, the risk of depression was higher among youth with a family to some extent or not at all when compared to those having a loving and affectionate relationship to a very great extent. While past research has explored this relationship, the magnitude of its association had not been assessed. Strong family support helps reduce depression risk by providing emotional warmth, stress coping resources, and boosting self-esteem.^31^ On the contrary, youth reporting only partial or no loving and affectionate relationships with family had lower odds of anxiety compared to those reporting very strong loving family relationships. The reasons for these present findings are unclear, as a loving relationship with family has been a protective factor for mental health.^32^ Further studies are needed to better understand this relationship.

 Evidence suggests that stable, healthy friendships are important for well-being and longevity.^33^ Having close friendships appears to protect against depression. In the event of life’s challenges, having a close friend to turn to may serve as a buffer or protective factor against negative outcomes. Friendships protect individuals partly by shaping how they respond to stress.^26^ Based on this evidence, promoting social networks, connections, and peer support among youth could be an important intervention to address the burden of depression in the study area.

 Spirituality has also been found to play a positive role in depression, either by facilitating recovery or providing protection against it. In the current study, spirituality was assessed through belief in God. Participants with strong beliefs in God were less likely to be depressed compared to those with neutral or weaker beliefs. Our findings align with previous research linking spirituality to positive emotions such as life satisfaction, well-being, hope, and purpose, which can counteract the negative emotions underlying depression. ^34^

 In the Kolar district, youth involved in physical violence were more prone to anxiety. A South African study similarly reported higher anxiety scores among adolescents exposed to violence.^35^ Whether the factors, circumstances, or context that led to violence among youth, or the aftermath of such violence, are responsible for the increased risk of anxiety is not clearly known and warrants further study. Youth with self-reported RTIs in the past 12 months were also at increased risk of anxiety, consistent with findings reported elsewhere.^36^ Post-traumatic stress, challenges in rehabilitation following injury, or the socioeconomic consequences of RTIs could have contributed to this increased risk. Furthermore, our study reaffirmed the established connection between smoking and anxiety.^37-39^ Similarly, youth who self-reported diagnosed health issues, particularly chronic conditions, were more likely to screen positive for anxiety. A systematic review has identified a strong link between anxiety and common medical conditions.^40^ Experiencing chronic health issues at a young age, the need for long-term care and support, and the socioeconomic implications of managing such chronic health issues may have increased the risk of anxiety among youth with health problems.

 Youth from KGF Taluka were found to have twice the odds of being screened positive for depression compared to those from Bangarpete Taluka in the Kolar district. This elevated risk among KGF youth may be attributed to factors such as poverty, lack of industrial development, harsh living conditions, insecure housing, limited income, and lack of opportunities, which are common in KGF Taluka. These findings highlight the need to prioritize mental health interventions for youth in KGF. The risk of screening positive for depression was higher among youth aged 26-30 years compared to other age groups. This aligns with existing literature, which suggests that depression becomes more common later in young adulthood.^41,42^ The elevated stress in this age group may be linked to increased work pressures, family responsibilities, and other demands of early adulthood, all of which can contribute to higher rates of depression.

 The present study has several strengths. The large sample size of 5,072 individuals, interviewed at the district level in the 15-30 years age group and covering various health and health-impacting behaviors using standardized tools and methods, enabled us to comprehensively identify factors associated with anxiety and depression, specifically among youth.

 However, the study is not without limitations. Firstly, the cross-sectional nature of the study limits the ability to establish temporality in the observed associations between anxiety/depression and the identified risk factors. Moreover, the stigma surrounding mental health issues may have discouraged some participants from disclosing information, thereby influencing the observed associations. However, to reduce social desirability bias, field data collectors were well trained in data collection, and most interviews were conducted privately with confidentiality assured.

**Highlights**

- This secondary analysis uniquely examines associations between diverse health behaviors of youth with depression and anxiety in the Indian context.
- Excessive computer/mobile use, poor self-reported mental health, weak family relationships, and sleep problems were linked to a higher likelihood of depression and anxiety.
- Serious family conflicts, injury due to physical violence, road traffic injuries (RTIs), and non-suicidal self-harm were associated with increased depression risk.
- Suicide attempts were positively associated with anxiety, whereas supportive friendships and belief in God were protective against it.
- These identified risk and protective factors can guide healthcare providers and policymakers in developing tailored interventions.

## Conclusion

 This study highlights that select sociodemographic characteristics, health-impacting behaviors, and health issues among youth were significantly associated with being screened positive for anxiety and/or depression. While population-level mental health interventions are essential, targeted interventions focusing on high-risk groups or individuals are equally crucial from a public health perspective. The risk factors for depression and anxiety identified in this study can enable healthcare providers and policymakers to develop tailored interventions. Further research is recommended to firmly establish the associations observed in the present study.

## Acknowledgments

 The authors extend their sincere gratitude to Dr. Girish N. Rao, Dr. Senthil Amudhan, and Dr. Gopalkrishna Gururaj for their invaluable guidance, support, and assistance in conducting the Kolar Youth Health Survey. They are also deeply grateful to the data collectors and participants, whose involvement and contributions made this study possible.

## Competing Interests

 The authors declared no potential conflict of interests with respect to the research, authorship, and/or publication of this article.

## Ethical Approval

 Ethical approval for the present study was obtained from the Institutional Ethics Committee at NIMHANS (Letter No. NIMH/DO/IEC (BS & NS DIV)/2022). The primary study, *Kolar Youth Health Survey*, had earlier received approval from the NIMHANS Ethics Committee (Letter No. NIMHANS/20TH IEC (BS & NS DIV.)/2019, dated 03/09/2019).

## Funding

 The authors received no financial support for the research, authorship, and/or publication of this article.

## References

[1] GBD 2019 Diseases and Injuries Collaborators (2020). Global burden of 369 diseases and injuries in 204 countries and territories, 1990-2019: a systematic analysis for the Global Burden of Disease Study 2019. Lancet.

[2] Gururaj G, Varghese M, Benegal V, Rao GN, Pathak K, Singh LK, et al. National Mental Health Survey of India, 2015-16 Prevalence, Pattern and Outcomes. National Institute of Mental Health and Neuro Sciences; 2016.

[3] World Health Organization (WHO). Depression and Other Common Mental Disorders: Global Health Estimates. WHO; 2017.

[4] McMakin DL, Alfano CA (2015). Sleep and anxiety in late childhood and early adolescence. Curr Opin Psychiatry.

[5] Grover S, Raju VV, Sharma A, Shah R (2019). Depression in children and adolescents: a review of Indian studies. Indian J Psychol Med.

[6] Stiglic N, Viner RM (2019). Effects of screentime on the health and well-being of children and adolescents: a systematic review of reviews. BMJ Open.

[7] Nawi AM, Ismail R, Ibrahim F, Hassan MR, Abdul Manaf MR, Amit N (2021). Risk and protective factors of drug abuse among adolescents: a systematic review. BMC Public Health.

[8] Upadhyay D, Agrawal M. Youth, Mental Health, Well-Being and Development Issues. Lucknow: Amity Institute of Behavioral and Allied Sciences, Amity University Lucknow Campus; 2017.

[9] Hidaka BH (2012). Depression as a disease of modernity: explanations for increasing prevalence. J Affect Disord.

[10] Kroenke K, Spitzer RL, Williams JB (2001). The PHQ-9: validity of a brief depression severity measure. J Gen Intern Med.

[11] Spitzer RL, Kroenke K, Williams JB, Löwe B (2006). A brief measure for assessing generalized anxiety disorder: the GAD-7. Arch Intern Med.

[12] Garber J, Weersing VR (2010). Comorbidity of anxiety and depression in youth: implications for treatment and prevention. Clin Psychol (New York).

[13] Mathew AR, Pettit JW, Lewinsohn PM, Seeley JR, Roberts RE (2011). Co-morbidity between major depressive disorder and anxiety disorders: shared etiology or direct causation?. Psychol Med.

[14] Blanco C, Rubio JM, Wall M, Secades-Villa R, Beesdo-Baum K, Wang S (2014). The latent structure and comorbidity patterns of generalized anxiety disorder and major depressive disorder: a national study. Depress Anxiety.

[15] Gollust SE, Eisenberg D, Golberstein E (2008). Prevalence and correlates of self-injury among university students. J Am Coll Health.

[16] Nagy LM, Shanahan ML, Baer RA (2021). An experimental investigation of the effects of self-criticism and self-compassion on implicit associations with non-suicidal self-injury. Behav Res Ther.

[17] Ahmed HU, Hossain MD, Aftab A, Soron TR, Alam MT, Chowdhury MW (2017). Suicide and depression in the World Health Organization South-East Asia region: a systematic review. WHO South East Asia J Public Health.

[18] Khalaf AM, Alubied AA, Khalaf AM, Rifaey AA (2023). The impact of social media on the mental health of adolescents and young adults: a systematic review. Cureus.

[19] Twenge JM, Campbell WK (2018). Associations between screen time and lower psychological well-being among children and adolescents: evidence from a population-based study. Prev Med Rep.

[20] Khouja JN, Munafò MR, Tilling K, Wiles NJ, Joinson C, Etchells PJ (2019). Is screen time associated with anxiety or depression in young people? Results from a UK birth cohort. BMC Public Health.

[21] El-Sayed Desouky D, Abu-Zaid H (2020). Mobile phone use pattern and addiction in relation to depression and anxiety. East Mediterr Health J.

[22] Alhassan AA, Alqadhib EM, Taha NW, Alahmari RA, Salam M, Almutairi AF (2018). The relationship between addiction to smartphone usage and depression among adults: a cross sectional study. BMC Psychiatry.

[23] Narmandakh A, Roest AM, Jonge P, Oldehinkel AJ (2020). The bidirectional association between sleep problems and anxiety symptoms in adolescents: a TRAILS report. Sleep Med.

[24] Lovato N, Gradisar M (2014). A meta-analysis and model of the relationship between sleep and depression in adolescents: recommendations for future research and clinical practice. Sleep Med Rev.

[25] Marino C, Andrade B, Campisi SC, Wong M, Zhao H, Jing X (2021). Association between disturbed sleep and depression in children and youths: a systematic review and meta-analysis of cohort studies. JAMA Netw Open.

[26] Borst JB. A Systematic Review of the Effects of Family Conflict: Focusing on Divorce, Infidelity, and Attachment Style [dissertation]. Minnesota: University of St. Thomas; 2015.

[27] Adare AA, Zhang Y, Hu Y, Wang Z (2021). Relationship between parental marital conflict and social anxiety symptoms of Chinese college students: mediation effect of attachment. Front Psychol.

[28] Grundström J, Konttinen H, Berg N, Kiviruusu O (2021). Associations between relationship status and mental well-being in different life phases from young to middle adulthood. SSM Popul Health.

[29] Hald GM, Ciprić A, Sander S, Strizzi JM (2022). Anxiety, depression and associated factors among recently divorced individuals. J Ment Health.

[30] Amato PR (2010). Research on divorce: continuing trends and new developments. J Marriage Fam.

[31] Thomas PA, Liu H, Umberson D (2017). Family relationships and well-being. Innov Aging.

[32] Chen P, Harris KM (2019). Association of positive family relationships with mental health trajectories from adolescence to midlife. JAMA Pediatr.

[33] Song I, Kwon JW, Jeon SM (2023). The relative importance of friendship to happiness increases with age. PLoS One.

[34] Bonelli R, Dew RE, Koenig HG, Rosmarin DH, Vasegh S (2012). Religious and spiritual factors in depression: review and integration of the research. Depress Res Treat.

[35] Stansfeld SA, Rothon C, Das-Munshi J, Mathews C, Adams A, Clark C (2017). Exposure to violence and mental health of adolescents: South African Health and Well-being Study. BJPsych Open.

[36] van der Vlegel M, Polinder S, Toet H, Panneman MJ, Geraerds A, Haagsma JA (2022). Anxiety, depression and post-traumatic stress symptoms among injury patients and the association with outcome after injury. Eur J Psychotraumatol.

[37] Mykletun A, Overland S, Aarø LE, Liabø HM, Stewart R (2008). Smoking in relation to anxiety and depression: evidence from a large population survey: the HUNT study. Eur Psychiatry.

[38] Goodwin RD, Zvolensky MJ, Keyes KM (2008). Nicotine dependence and mental disorders among adults in the USA: evaluating the role of the mode of administration. Psychol Med.

[39] Moylan S, Jacka FN, Pasco JA, Berk M (2012). Cigarette smoking, nicotine dependence and anxiety disorders: a systematic review of population-based, epidemiological studies. BMC Med.

[40] Katon W, Lin EH, Kroenke K (2007). The association of depression and anxiety with medical symptom burden in patients with chronic medical illness. Gen Hosp Psychiatry.

[41] Szymkowicz SM, Gerlach AR, Homiack D, Taylor WD (2023). Biological factors influencing depression in later life: role of aging processes and treatment implications. Transl Psychiatry.

[42] Schaakxs R, Comijs HC, van der Mast RC, Schoevers RA, Beekman ATF, Penninx B (2017). Risk factors for depression: differential across age?. Am J Geriatr Psychiatry.

